# The Effect of Deflazacort Treatment on the Functioning of Skeletal Muscle Mitochondria in Duchenne Muscular Dystrophy

**DOI:** 10.3390/ijms21228763

**Published:** 2020-11-19

**Authors:** Mikhail V. Dubinin, Eugeny Yu. Talanov, Kirill S. Tenkov, Vlada S. Starinets, Natalia V. Belosludtseva, Konstantin N. Belosludtsev

**Affiliations:** 1 Department of Biochemistry, Cell Biology and Microbiology, Mari State University, pl. Lenina 1, 424001 Yoshkar-Ola, Russia; kirill.tenkove@gmail.com (K.S.T.); vlastar@list.ru (V.S.S.); bekonik@gmail.com (K.N.B.); 2 Laboratory of Mitochondrial Transport, Institute of Theoretical and Experimental Biophysics, Russian Academy of Sciences, Institutskaya 3, 142290 Pushchino, Russia; evg-talanov@yandex.ru (E.Y.T.); nata.imagination@gmail.com (N.V.B.); 3 Biophotonics Center, Prokhorov General Physics Institute, Russian Academy of Sciences, Vavilov st. 38, 119991 Moscow, Russia

**Keywords:** Duchenne muscular dystrophy, skeletal muscle, deflazacort, mitochondria, Ca^2+^ uniporter, mitochondrial permeability transition

## Abstract

Duchenne muscular dystrophy (DMD) is a severe hereditary disease caused by a lack of dystrophin, a protein essential for myocyte integrity. Mitochondrial dysfunction is reportedly responsible for DMD. This study examines the effect of glucocorticoid deflazacort on the functioning of the skeletal-muscle mitochondria of dystrophin-deficient *mdx* mice and WT animals. Deflazacort administration was found to improve mitochondrial respiration of *mdx* mice due to an increase in the level of ETC complexes (complexes III and IV and ATP synthase), which may contribute to the normalization of ATP levels in the skeletal muscle of *mdx* animals. Deflazacort treatment improved the rate of Ca^2+^ uniport in the skeletal muscle mitochondria of *mdx* mice, presumably by affecting the subunit composition of the calcium uniporter of organelles. At the same time, deflazacort was found to reduce the resistance of skeletal mitochondria to MPT pore opening, which may be associated with a change in the level of ANT2 and CypD. In this case, deflazacort also affected the mitochondria of WT mice. The paper discusses the mechanisms underlying the effect of deflazacort on the functioning of mitochondria and contributing to the improvement of the muscular function of *mdx* mice.

## 1. Introduction

Duchenne muscular dystrophy (DMD) is the most prevalent muscular dystrophy afflicting ~1 in 3500–5000 live born males [1,2]. Regarded as a debilitating and fatal skeletal muscle disease, DMD is characterised by muscular weakness, exercise intolerance, and progressive deterioration of skeletal muscle. DMD is caused by large deletions and small forms of other mutations (deletions, duplications, insertions, and substitutions) in the dystrophin gene located on the short arm of the X-chromosome. The protein encoded by this gene is involved in the formation of a complex functional network (the so-called dystrophin-associated protein complex) which ensures the integrity and rigidity of muscle fibers. However, dystrophin deficiency and secondary reduction of these glycoproteins [3] render the myoctes and their fibers more susceptible to damage as they become structurally unstable and exceedingly porous to the extracellular environment. As a result, excessive Ca^2+^ influx, poor Ca^2+^ handling, activation of proteases/lipases, and mitochondrial Ca^2+^ overload precede muscle degeneration [4,5,6,7]. While the exact underlying pathomechanisms still require more research, over time, and as regeneration fails, fatty and connective tissue replacement culminates in non-functional muscle tissue [7].

According to several reports, DMD is characterised by a systemic metabolic impairment, which is central to the etiology of the disease. Indeed, DMD is accompanied by a significant deficiency of the glycolysis [8,9,10] and purine nucleotide cycle [11,12] enzymes, as well as the enzymes involving the tricarboxylic acid (TCA) cycle [10,13] and the electron transport chain (ETC) [14,15,16]. These changes lead to reduced ATP levels in skeletal muscle in both DMD patients and *mdx* models [17,18,19,20]. This, in turn, results in dysfunction of the contractile apparatus, leading to reduced muscular strength, dysregulation of intracellular Ca^2+^ buffering, loss of homeostasis, and Ca^2+^-induced degeneration.

DMD-induced dysregulation of intracellular Ca^2+^ homeostasis is hypothesized to associated with the dysfunction of mitochondria [14,15,16,21,22,23]. It was found that DMD results in a decrease in the efficiency of transport and accumulation of Ca^2+^ in mitochondria, which develops in the skeletal muscle already in the early stages of postnatal development and is accompanied by a decrease in the rate of mitochondrial oxidative phosphorylation, as well as a decrease in resistance to the mitochondrial permeability transition (MPT) pore opening due to ROS overproduction and depolarization of organelles [14,15,16,23,24,25]. Recently, we showed that the dysregulation of calcium homeostasis in skeletal muscle mitochondria of *mdx* mice may be due to rearrangements in the systems responsible for the transport of calcium ions and MPT pore opening [26].

**Figure 1:**
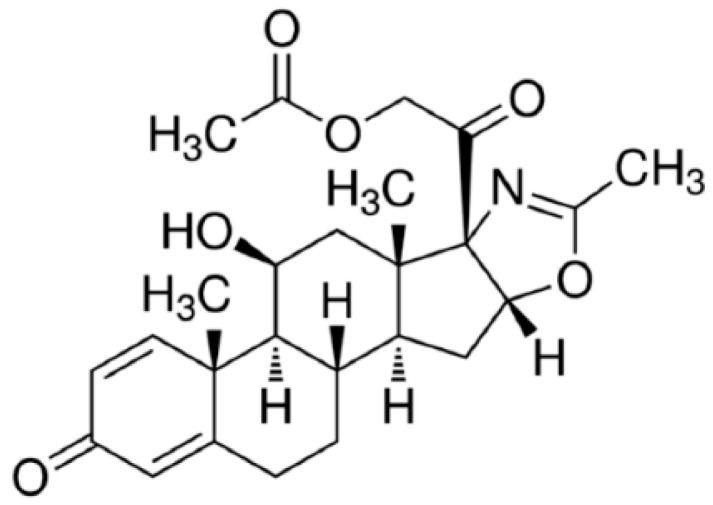
Chemical structure of deflazacort.

Currently, one of the amenable disease interventions available to DMD patients is through the administration of a subclass of glucocorticoids, specifically prednisone and its oxazoline derivative deflazacort (Dfz, Figure 1). Some other experimental trials are ongoing, such as myostatin inhibition [27] and/or Sarepta’s micro-dystrophin gene therapy [28], which show promising results. However, currently Dfz is the first drug approved by the United States Food and Drug Administration (FDA) for the management of DMD. It is known that Dfz in the form of an active metabolite, 21-desacetyldeflazacort, is able to inhibit the release of cytokines by inhibiting the proliferation of CD4+ lymphocytes and at the same time increasing the number of CD8+ cells, which helps to suppress the immune response and inflammatory processes in muscle tissue [29,30]. Dfz-based therapy prolongs ambulation by 2–5 years and modestly improves muscle strength and cardiopulmonary function [30] but is often associated with significant side effects such as a Cushingoid appearance, erythema, hirsutism, increased weight, nasopharyngitis, delayed puberty, cataracts, and vertebral fractures [31,32,33]. Glucocorticoids also can disrupt the expression of genes involved in muscle degradation and regeneration [34] leading to chronic myopathies that contribute to proximal muscle weakness [35]. In particular, long-term use of glucocorticoids has been shown to induce muscle atrophy due to inhibition of muscle protein synthesis [36,37].

In this work, we first evaluated the effect of Dfz administration on the functional activity of skeletal muscle mitochondria of dystrophin-deficient C57BL/10ScSn-Dmd*mdx* line (*mdx* mice) and the wild-type C57BL/10 line (wild type mice). The results obtained indicate that, on the one hand, deflazacort administration improves mitochondrial respiration of *mdx* mice and the ability of organelles to accumulate Ca^2+^, which may contribute to increase the ATP level in the skeletal muscles of these animals. On the other hand, deflazacort reduces the resistance of the skeletal muscle mitochondria of the studied groups of mice to the opening of Ca^2+^-dependent MPT pore. The observed effects of deflazacort may be due to rearrangements in the mitochondrial proteome (ETC complexes, calcium uniporter, putative MPT-pore proteins) and, apparently, contribute to the improvement of the muscle function of *mdx* mice.

## 2. Results

### 2.1. Effect of Deflazacort Administration on Respiration and Oxidative Phosphorylation of Skeletal Muscle Mitochondria of Dystrophin-Deficient and WT Mice

**Figure 2:**
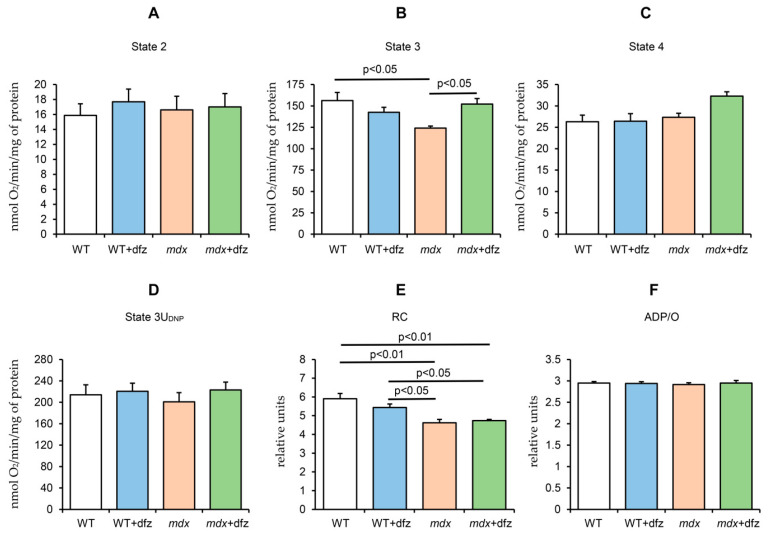
Effect of deflazacort administration on glutamate/malate-fueled respiration of skeletal-muscle mitochondria of wild-type and *mdx* mice in different functional states (**A**–**D**) and on the parameters of respiratory control (**E**) and ADP/O (**F**). Medium composition: 120 mM KCl, 5 mM NaH_2_PO_4_, and 10 mM HEPES-KOH buffer (pH 7.4) Respiration of mitochondria was fueled by 2.5 mM glutamate + 2.5 mM malate. Respiration of mitochondria in state 3 was initiated by 200 μM ADP. The rate of uncoupled respiration was measured in the presence of 50 μM DNP (state 3U_DNP_). The data are presented as means ± SEM (*n* = 4).

DMD is known to be associated with significant dysfunction of skeletal muscle mitochondria due to decreased oxidative phosphorylation efficiency and oxidative stress. Indeed, one can see that mitochondria of dystrophin-deficient mice show a significant decrease in the rate of ADP-stimulated respiration (state 3) and respiratory control ratio compared to WT animals (Figure 2). Deflazacort administration increases the respiration rate of *mdx* mice mitochondria in state 3 by 23% and has no effect on other parameters of mitochondrial respiration and oxidative phosphorylation in both WT mice and dystrophin-deficient animals.

**Figure 3:**
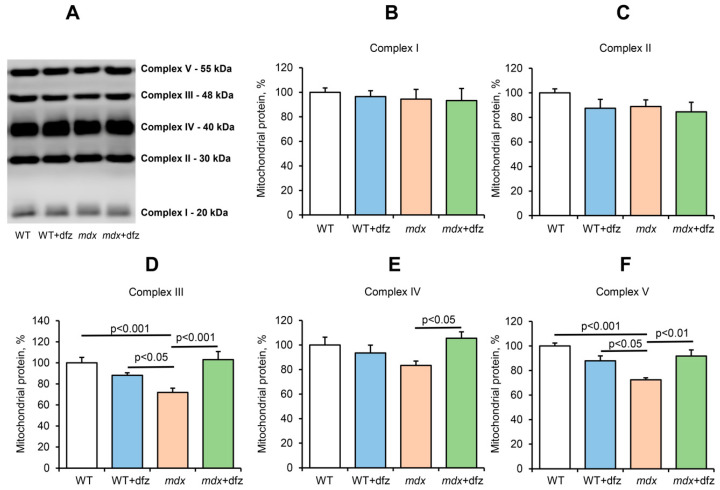
Levels of the proteins of mitochondrial respiratory chain complexes. Data of Western blot analysis (**A**). Relative contents of Complex I (**B**), Complex II (**C**), Complex III (**D**), Complex IV (**E**), and Complex V (ATP synthase, **F**). The data are presented as means ± SEM (*n* = 6).

Inhibition of mitochondrial respiration in DMD animals may be associated with a decrease in the level of some complexes of the respiratory chain of organelles, as well as ATP synthase [14,15,16,26]. In this work, we examined the effect of deflazacort administration on the level of proteins that form the complexes of the respiratory chain of mitochondria in the skeletal muscle of the studied animal groups. One can see that the level of complexes I, II, and IV in the mitochondria of *mdx* mice does not change compared to control animals (Figure 3). At the same time, mitochondria of dystrophin-deficient animals show a significant decrease in the level of complex III and ATP synthase. Deflazacort does not affect the level of complexes I, II, and IV in the mitochondria of the studied animals. However, treatment with this glucocorticoid normalizes the level of complex III and ATP synthase in *mdx* mice mitochondria to the level of WT animals. In addition, deflazacort administration leads to an increase in the level of complex IV in the mitochondria of *mdx* mice compared to control dystrophin-deficient animals.

**Figure 4:**
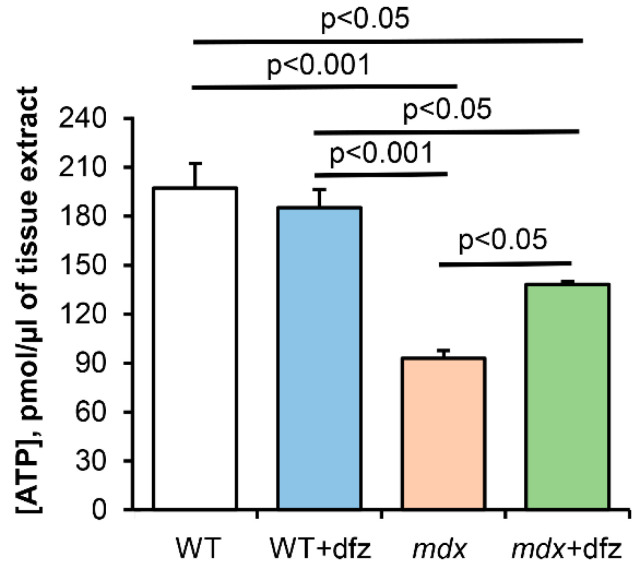
The level of ATP in the extract of skeletal muscle tissue of mice after four weeks of treatment with deflazacort. The data are presented as means ± SEM (*n* = 3).

Mitochondrial dysfunction contributes significantly to lower ATP levels in skeletal muscle. Indeed, one can see that the skeletal muscles of dystrophin-deficient animals show more than a twofold decrease in the ATP level compared to WT mice (Figure 4). In this case, treatment of *mdx* mice with deflazacort leads to a significant increase in the ATP level compared to control dystrophin-deficient animals. However, it should be noted that these data will require confirmation in a larger cohort.

### 2.2. Effect of Deflazacort Administration on Ca^2+^ Uniport in the Skeletal-Muscle Mitochondria of Dystrophin-Deficient and WT Mice

**Figure 5:**
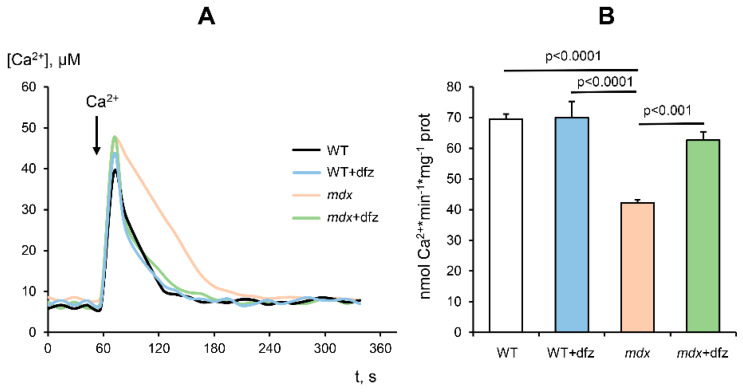
The dynamics of Ca^2+^ uptake by skeletal-muscle mitochondria of studied groups of mice (**A**). Incubation medium: 210 mM mannitol, 70 mM sucrose, 2.5 mM glutamate, 1 mM KH_2_PO_4_, 50 μM arsenazo III, 10 µM EGTA, 2.5 mM malate, 1 µM cyclosporin A, 10 mM Hepes/KOH, pH 7.4. Additions: skeletal-muscle mitochondria (0.4–0.5 mg/mL), 50 μM CaCl_2_. The figure shows typical measurements conducted at the same time on the same mitochondrial preparation. The results were similar in five independent experiments. (**B**) Rate of Ca^2+^ uniport in the skeletal-muscle mitochondria of studied groups of mice. The data are presented as means ± SEM (*n* = 5).

The degradation of skeletal muscles that occurs in Duchenne muscular dystrophy is caused, among other things, by significant dysregulation of mitochondrial calcium homeostasis. As we showed earlier [26] and confirmed here, skeletal muscle mitochondria of dystrophin-deficient mice show a significant decrease in the rate of Ca^2+^ uptake in comparison with mitochondria of wild-type mice (Figure 5). In this case, deflazacort treatment normalizes the rate of Ca^2+^ uniport in skeletal muscle mitochondria of dystrophin-deficient animals to the level of wild-type ones. It should also be noted that this glucocorticoid has no effect on the rate of Ca^2+^ uptake in the mitochondria of WT mice.

**Figure 6:**
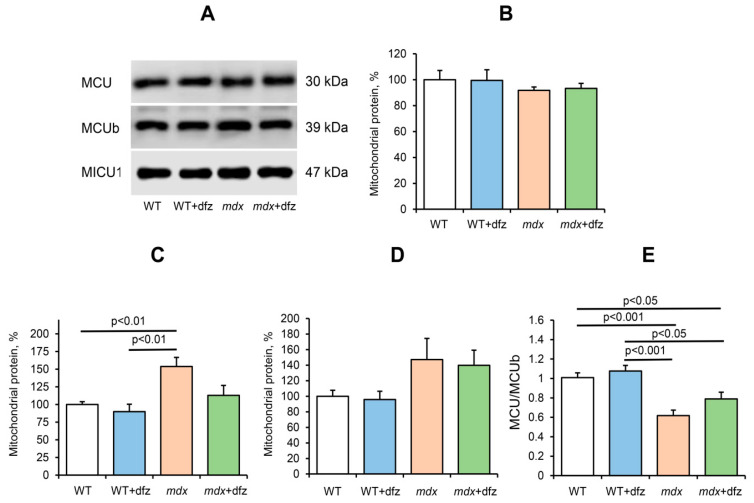
Levels of the proteins of the mitochondrial Ca^2+^ uniporter in the skeletal-muscle mitochondria of the studied groups of mice. Data of Western blot analysis (**A**). Relative contents of MCU (**B**), MCUb (**C**), MICU1 (**D**) subunits. MCU/MCUb ratio in mitochondria of studied groups of mice (**E**). The data are presented as means ± SEM (*n* = 6).

The uptake of Ca^2+^ by mitochondria is known to be mediated by Ca^2+^ uniporter, a complex of the inner mitochondrial membrane, which includes channel subunits (MCU and MCUb), regulatory subunits (MICU1-2, EMRE, MCUR1), and a number of other proteins [38,39,40]. It is considered that the contents of MCU, MCUb, and MICU1 and their stoichiometry can predominantly regulate mitochondrial Ca^2+^ transport in accordance with physiological needs [38,41,42,43]. Moreover, a number of pathologies reveal the effect of the ratio of these subunits on the parameters of calcium transport in mitochondria [44,45,46,47]. Indeed, we found a similar pattern in the case of DMD [26,48]. We have found that skeletal muscle mitochondria of dystrophin-deficient mice show an increase in the level of MCUb (inactive channel subunit of the Ca^2+^ uniporter), which is accompanied by a significant decrease in the MCU/MCUb ratio. The data obtained in this work show that a similar trend persists in 7-week-old mice (Figure 6). Here, we examined the effect of deflazacort administration on the level of the main MCUC subunits, which determine the kinetics of Ca^2+^ uniport in the skeletal muscle mitochondria of experimental animal groups (MCU and MCUb channel subunits and regulatory MICU1 subunit). One can see that the deflazacort treatment does not affect the level of the MCU subunit in the mitochondria of the studied animals, but shows a tendency towards a decrease in the level of the dominant negative MCUb subunit in the skeletal muscle mitochondria of *mdx* mice (Figure 6). However, this is not accompanied by a significant change in the MCU/MCUb ratio in mitochondria of deflazacort-treated *mdx* mice compared to control dystrophin-deficient animals (Figure 6C). It should be noted that deflazacort does not significantly affect the level of the regulatory MICU1 subunit in the skeletal muscle mitochondria of the studied animals.

### 2.3. Effect of Deflazacort Administration on MPT Pore Opening in the Skeletal-Muscle Mitochondria of Dystrophin-Deficient and WT Mice

**Figure 7:**
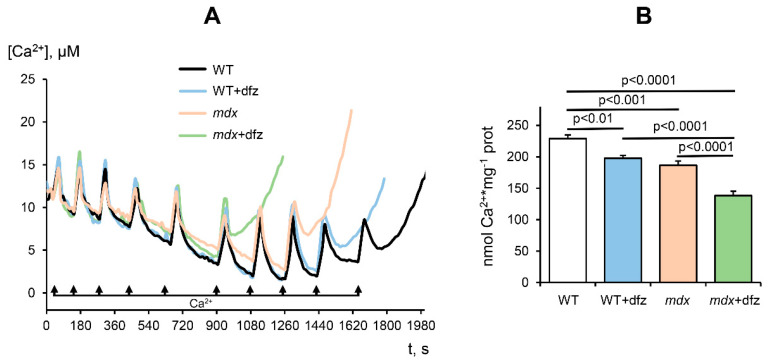
Induction of the Ca^2+^-dependent MPT pore in the skeletal-muscle mitochondria of the studied groups of mice. Uptake of sequential Ca^2+^ additions by the skeletal-muscle mitochondria (**A**). Incubation medium: 210 mM mannitol, 70 mM sucrose, 2.5 mM glutamate, 1 mM KH_2_PO_4_, 50 μM arsenazo III, 10 μM EGTA, 2.5 mM malate, 10 mM Hepes/KOH (pH 7.4). Additions: skeletal-muscle mitochondria (0.45 mg/mL), 10 μM CaCl_2_. The figure shows traces of a typical experiment conducted at the same time on the same mitochondrial preparation. Similar results were obtained in five independent experiments. Ca^2+^ capacity of the skeletal-muscle mitochondria of studied groups of mice (**B**). The data are presented as means ± SEM (*n* = 5).

Pathological processes in the muscle tissue of *mdx* mice have been shown to be associated with calcium overload of mitochondria and MPT pore opening sensitive to a specific inhibitor CsA or its analogue alisporivir [49]. Mitochondrial resistance to MPT pore opening can be assessed by measuring the Ca^2+^ capacity of the organelles—the maximal amount of Ca^2+^ that can be accumulated in the matrix without subsequent induction of MPT [50]. Skeletal muscle mitochondria in *mdx* mice are characterized by a decrease in calcium capacity compared to mitochondria of WT mice (Figure 7), which is consistent with previously obtained data [26]. In this work, we also evaluated the effect of deflazacort on the calcium capacity of the skeletal muscle mitochondria of the studied animal groups. Figure 7 shows that for mitochondria of Dfz-treated *mdx* mice, the number of successive Ca^2+^ additions (and, therefore, the threshold pore-opening Ca^2+^ concentration) was lower compared with the control dystrophin-deficient animals. Moreover, this glucocorticoid has a similar effect on the mitochondria of WT mice. Thus, it can be assumed that deflazacort reduces the resistance of mitochondria to the opening of the MPT pore.

**Figure 8:**
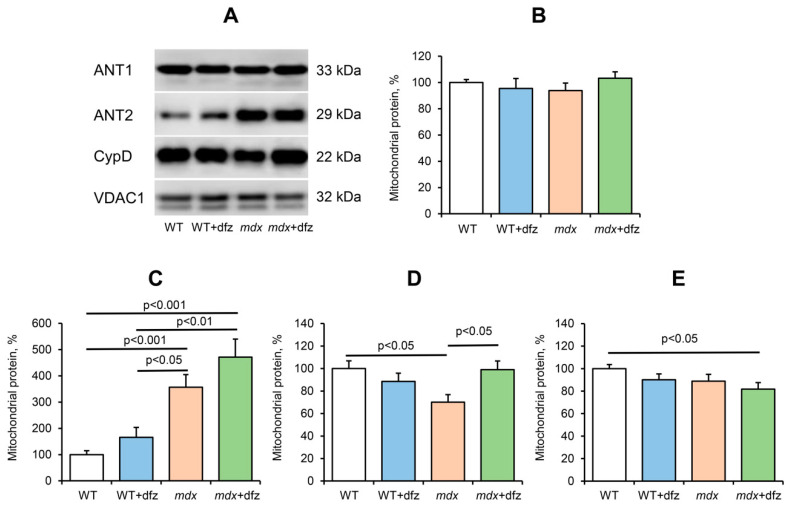
Levels of putative components of the MPT pore in the skeletal-muscle mitochondria of the studied groups of mice. Data of Western blot analysis (**A**). Relative contents of MPT-related proteins: ANT1 (**B**), ANT2 (**C**), CypD (**D**), VDAC1 (**E**). The data are presented as means ± SEM (*n* = 6).

Currently, the MPT pore is believed to be a mega-channel, penetrating both the outer and inner membranes of mitochondria. Currently, adenine nucleotide translocator, ATP synthase, cyclophilin D, and VDAC are considered to be essential components of the pore apparatus [51,52,53,54]. We previously showed that a decrease in the resistance of the skeletal muscle mitochondria of *mdx* mice may be due to a significant increase in the level of ANT2, which, most likely, forms the pore channel in skeletal muscle mitochondria in this pathology [26]. Indeed, one can see that an elevated level of this protein persists in the mitochondria of 7-week-old *mdx* mice (Figure 8). In this case, deflazacort administration tends to increase the level of ANT2 in mitochondria of both *mdx* mice and WT animals. The ANT1 level does not change. Along with this, we noted an increase in the level of ATP synthase (Complex V) in the mitochondria of *mdx* mice (Figure 3), which is also able to form the MPT pore channel, as well as a tendency to normalize the level of cyclophilin D to the level of WT mice (Figure 8). In addition, one can see that deflazacort administration shows a weak trend towards a reduction in the level of VDAC1 in the mitochondria of both studied animal groups.

### 2.4. Deflazacort Improves the Muscle Function of Dystrophin-Deficient Mice

**Figure 9:**
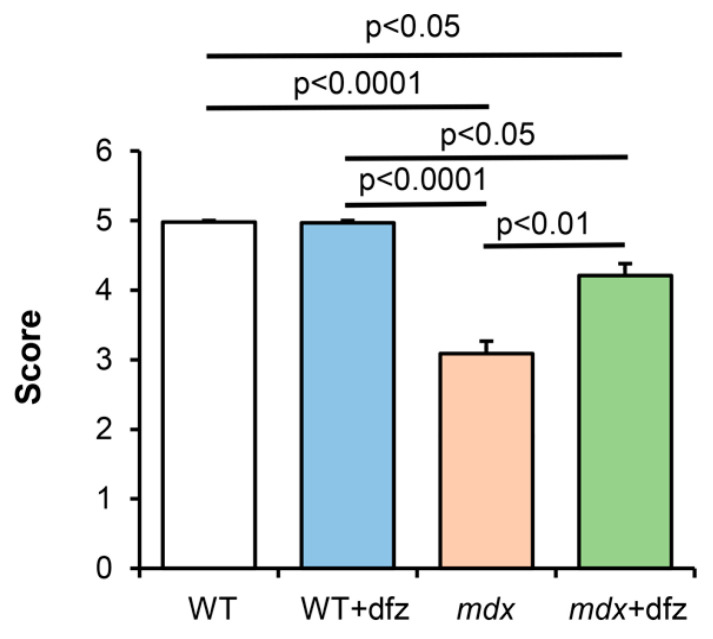
The results of a wire hang test, reflecting muscle function and endurance of the studied groups of mice. The data are presented as means ± SEM (*n* = 9).

In the last part of this work, we examined the effect of deflazacort administration on the muscle function of the studied animal groups. Figure 9 shows the results of a physiological test, which indicate a strong decrease in the endurance of dystrophin-deficient animals compared to WT mice. In this case, deflazacort-treated *mdx* mice showed a significant improvement in muscle function compared to control *mdx* mice. This is consistent with the data of other groups, indicating the beneficial effect of this glucocorticoid on the functional state of muscles in Duchenne dystrophy [36,55]. It should be noted that this therapeutic agent had no effect on the endurance of WT mice.

## 3. Discussion

Mitochondrial dysfunction is considered one of the reasons contributing to the progression of DMD. One of the ways to manage DMD is the use of corticosteroids and, in particular, deflazacort approved by the FDA for the treatment of this pathology in 2017. This drug showed good results in the management of Duchenne dystrophy in both humans and model animals, both when used alone and in combination with other drugs, in particular, myostatin inhibitors [37]. However, the mechanisms of its action remain poorly understood. In this work, we examined the effect of deflazacort administration (at a therapeutic dose of 1 mg/kg body weight) on the functional activity of skeletal muscle mitochondria in DMD mice. One can see that deflazacort-treated *mdx* mice show a significant increase in the rate of ADP-stimulated respiration of organelles compared to control dystrophin-deficient animals (Figure 2B). We suggest that an improvement in mitochondrial respiration in this case may be due to an increase in the level of the mitochondrial respiratory chain complexes of *mdx* animals (complexes III and IV and ATP synthase) (Figure 3). However, this effect of deflazacort was not accompanied by an increase in respiratory control ratio and ADP/O (Figure 2E,F). It is known that glucocorticoids can have an uncoupling effect on mitochondria, which is manifested in an increase in the state 4 respiration rate [56]. The dfz-treated *mdx* mice mitochondria also show a similar trend (Figure 2C). We assume that the uncoupling effect of deflazacort and its metabolic product on the respiration of organelles in state 4 result in no changes in the respiratory control ratio and ADP/O. It should be noted that previously similar effects were revealed for another corticosteroid, prednisone, which also improved mitochondrial respiratory function [57,58]. One could assume that a deflazacort-induced increase in the functional activity of organelles can also lead to a tendency to normalize ATP level in the skeletal muscles of *mdx* animals (Figure 4). On the other hand, given the anti-inflammatory activity of this agent [29,30], it can be assumed that a decrease in the intensity of inflammatory processes in the skeletal muscle of deflazacort-treated *mdx* mice, and, accordingly, in the intensity of regeneration, can also reduce the ATP demand of muscle cells, as well as mitigate the dysfunction of muscle tissue, cells, and organelles. In addition, it is necessary to note the possible effect of this agent on the activity of glycolysis and TCA cycle enzymes.

It is known that *mdx* animals are characterized by severe dysregulation of mitochondrial Ca^2+^ homeostasis [26]. Indeed, the skeletal muscle mitochondria of *mdx* mice show a significantly lower rate of calcium uptake compared to WT mice (Figure 5). On the one hand, this may be due to a decrease in the functional activity of organelles, and on the other hand, it may be associated with rearrangements in mitochondrial Ca^2+^-transport systems. The main mechanism of Ca^2+^ uptake in mitochondria is an energy-dependent uniport carried out by a uniporter complex located in the inner membrane of organelles. The mitochondrial Ca^2+^ uniporter complex (MCUC) is considered to consist of two transmembrane channel subunits MCU and MCUb (the latter has no channel activity) and regulatory subunits MICU1-2, EMRE, etc. We have previously shown that an increase in the level of the dominant negative MCUb subunit (and, correspondingly, a decrease in the MCU/MCUb ratio) can lead to a decrease in the Ca^2+^ uniport rate in the skeletal muscle mitochondria of *mdx* mice [26]. Indeed, these data are confirmed in the present work (Figure 6). In this case, it can be seen that deflazacort administration is accompanied by an improvement in the rate of Ca^2+^ uniport in the mitochondria of *mdx* mice (Figure 5) to the level of WT animals. Such an effect of deflazacort may be partially due to the influence on the expression of the MCUb subunit (Figure 6). This is in good agreement with literature data, which indicate the ability of glucocorticoids to modulate the activity of calcium carriers in the membranes of muscle cells, including mitochondrial membranes [59]. On the other hand, it should be noted that normalization of the Ca^2+^ uniport rate in mitochondria of *mdx* mice can also be associated with an improvement in the functioning of the electron transport chain of organelles and the efficiency of mitochondrial respiration.

It is well known that dysregulation of mitochondrial calcium transport in DMD is associated with a decrease in the resistance of skeletal muscle mitochondria to calcium-dependent MPT pore opening [16,26]. On the one hand, these effects may be due to the accumulation of ROS and oxidative stress development [16]. On the other hand, we have recently shown that proteins of the inner membrane capable of forming the MPT pore channel play an important role in reducing the resistance of mitochondria of *mdx* mice to pore opening. In particular, we found [26] that the skeletal muscle mitochondria of *mdx* mice show a significant increase in the level of ANT2, which normally does not occur in skeletal muscle mitochondria and can form the channel of the MPT pore [60]. One can see that deflazacort administration causes an increase in the level of ANT2 in the mitochondria of *mdx* mice, and this trend is also observed in the mitochondria of WT animals (Figure 8). Moreover, as shown above, skeletal muscle mitochondria in deflazacort-treated *mdx* mice show an increase in the level of ATP synthase, which is also able to form the channel of the MPT pore, as well as an increase in the level of the regulatory protein cyclophilin D (CypD). One can see that the level of ANT2, but not CypD and VDAC1, showed an inverse correlation with the resistance. This may be due to a certain tissue specificity in the involvement of MPT-related proteins in the formation of the pore and its regulation [61,62,63,64]. Indeed, it is known that the expression of the main regulatory protein CypD does not have a decisive effect on pore opening (unlike the situation when the gene of this protein is completely knocked out) [64]. On the other hand, the level of these multifunctional proteins can also have an important effect on the efficiency of oxidative phosphorylation of organelles and the development of dystrophy [65,66,67]. Finally, it should be noted that according to the latest data, the number of MPT pores and, accordingly, channel-forming proteins, does not exceed nine per mitochondria [68]. This, in turn, suggests the importance of other mechanisms of regulation of mitochondrial permeability, such as microviscosity of the membrane [48,69] and the level of inorganic phosphate [50]. Nevertheless, all these effects, apparently, can also contribute to a decrease in the resistance of mitochondria to MPT pore opening and the initiation of cell death. One could assume that such an effect of deflazacort may also underlie its many side effects. Indeed, it is known that prolonged use of this drug leads to muscle atrophy [36]. One could speculate that this could also be due to a decrease in organelle resistance to MPT pore opening and the induction of muscle cell death.

The molecular mechanisms of action of corticosteroids are still under discussion. It is suggested that the effect of deflazacort can be mediated by activation of glucocorticoid receptors, which leads to inhibition of the proinflammatory NF-*κ*B pathway initiated as a result of damaged muscle contraction [70]. Chronic activation of NF-*κ*B is a key driver of muscle degeneration and suppression of muscle regeneration in DMD [71,72], which occurs early in the disease process and precedes loss of muscle function. Recently, it was shown that the NF-*κ*B pathway is able to modulate the transcription of nuclear genes encoding calcium-transporting systems in muscle cells and the inhibition of this pathway improves the state of muscle tissue in DMD [73]. In addition, it should be noted that NF-*κ*B subunits were found in the mitochondria [74,75]. Mitochondrial NF-*κ*B are supposed to play an important role in the organelle dynamics [76], induction of apoptosis [77], and also to regulate the expression of mtDNA genes encoding ETC proteins [75,78]. In addition, most ETC subunits are nuclear DNA encoded and may be influenced by the NF-*κ*B pathway [79]. Thus, regulation of the activity of NF-*κ*B and other signaling pathways, including those directly associated with mitochondria, can play an important role in the modulating effect of deflazacort and other corticosteroids in DMD and contribute to the improvement of muscle tissue and animal’s endurance (Figure 9). Finally, as indicated above, steroid drugs, as hydrophobic compounds, can directly interact with membranes, including mitochondrial membranes, regulating their permeability and the activity of membrane proteins [59].

## 4. Materials and Methods

### 4.1. Animals

The mice used were C57BL10 mice (wild-type, WT) and dystrophin-deficient *mdx* (C57BL/10ScSn-mdx). All the animals were purchased from the Animal Breeding Facility, Branch of the Shemyakin and Ovchinnikov Institute of Bioorganic Chemistry, Russian Academy of Sciences, Russia (IBCh RAS Unique Research Device “Bio-model”, Pushchino, Russia). Upon arrival, mice were singly housed and given a minimum of 72 h to acclimatize before experiments were performed. All animals were provided access to standard chow and water ad libitum. The study with laboratory animals was carried out in accordance with the European Convention for the Protection of Vertebrates used for experimental and other purposes (Strasbourg, 1986) and the principles of the Helsinki Declaration (2000). All the protocols were approved by the Institute of Theoretical and Experimental Biophysics RAS Ethics Committee (Order No. 173/k of 03.10.2011, Protocol No. 11/2020 of 17.02.2020). Mitochondrial isolation was performed on fresh samples of skeletal muscle. The rest of the tissue was stored at −80 °C until analyzed.

### 4.2. Deflazacort Administration

**Figure 10:**
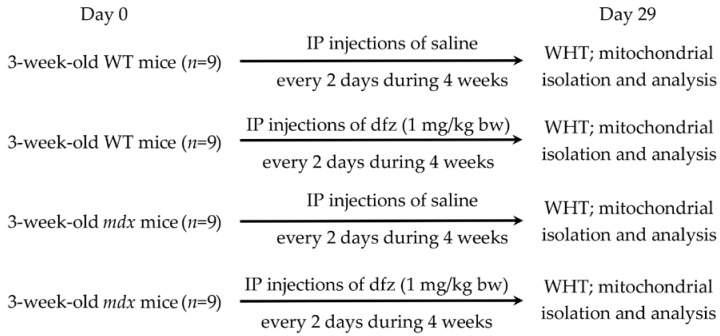
The experimental protocol of the study, IP: intraperitoneal; WHT: wire hanging test.

Using the experimental protocol shown in Figure 10, *mdx* and WT mice (three weeks old) were injected interperitoneally with deflazacort (Sigma-Aldrich, St. Louis, MO, USA) resuspended in sterile saline every three days for up to four weeks (1 mg/kg body weight). Sham-injected controls received saline alone. Mice were then killed and tissues were removed for analysis.

### 4.3. Physiological Test

For the assessment of muscle function and endurance of the animals, a wire-hang test was performed. A mouse was placed on a string (3 mm in diameter; 38 cm long; 49 cm above a padded surface), which the animal was holding with its front paws, and left to hang for 30 s. For the test, a soft padded surface was placed at the base of the apparatus to cushion any mice that fell off. The test scoring was performed as described by Deacon [80].

### 4.4. Mitochondria Isolation

Mitochondria were isolated from skeletal muscles (quadriceps of both hind limbs) using a convenient technique of differential centrifugation as previously described [81]. The isolation medium contained 67 mM sucrose, 50 mM KCl, 10 mM EDTA, 0.2% BSA, 50 mM Tris/HCl buffer, pH 7.4. The final suspension contained 30–40 mg of mitochondrial protein/mL, as determined by the Lowry method.

### 4.5. ATP Measurement in Skeletal Muscle

The level of ATP in the skeletal muscle tissue of mice was determined using a commercially available microplate assay kit (ATP Colorimetric/Fluorometric Assay Kit, cat.N. MAK190-1KT, Sigma-Aldrich, St. Louis, MO, USA). After decapitation of animals, the muscle tissue (quadriceps) was rapidly excised, immediately frozen in liquid nitrogen, and stored at −80 °C. A lysate for the assay was obtained from 10 mg of frozen homogenized tissue, which was blended with 100 µl of ATP assay buffer and then deproteinized using a 10kDa MWCO spin filter (Vivaspin 500 centrifugal concentrator, Sartorius AG, Goettingen, Germany) in accordance with the manufacturer’s recommendations. A 5 µL volume of lysate was used in the assay. The samples were processed as suggested by the manufacturer of the kit, using the appropriate standard concentrations of ATP to obtain a calibration curve. The fluorescence (λ_ex_ = 535/ λ_em_ = 587 nm) was measured on Tecan Spark 10M plate reader (Tecan, Männedorf, Switzerland). The concentration of ATP was expressed in pmol/µL of tissue extract.

### 4.6. Determination of Mitochondrial Respiration and Oxidative Phosphorylation

The rate of oxygen consumption was measured polarographically with a Clark-type gold electrode (O2k, OROBOROS Instruments, Innsbruck, Austria) at 25 °C under continuous stirring. The reaction medium contained 120 mM KCl, 5 mM NaH_2_PO_4_, 10 mM Hepes/KOH, pH 7.4. The concentrations of substrates and other reagents were as follows: 2.5 mM potassium malate, 2.5 mM potassium glutamate, 0.2 mM ADP, and 50 μM 2,4-dinitrophenol (DNP). The following were estimated: the mitochondrial respiration in a resting state (i.e., basal mitochondrial respiration in the presence of exogenous substrates or state 2), in state 3 (exogenous substrates plus ADP), in state 4 (after ADP exhaustion), and uncoupled state 3U_DNP_ in the presence of an uncoupler (2,4-dinitrophenol). The rates of substrate oxidation were expressed as nmol O_2_ × min^−1^ × mg^−1^ mitochondrial protein. Respiratory control (RC = state 3/state 4) and ADP/O ratios were determined according to Chance and Williams [82]. The concentration of mitochondrial protein was 0.5–1 mg/mL.

### 4.7. Uptake and Release of Mitochondrial Calcium, Determination of Ca^2+^ Retention by Mitochondria

The transport of Ca^2+^ across the inner mitochondrial membrane was monitored spectrophotometrically with an arsenazo III (2,2′-(1,8-Dihydroxy-3,6-disulfonaphthylene-2,7-bisazo)bisbenzenearsonic acid, 2,7-Bis(2-arsonophenylazo)chromotropic acid) indicator at 675–685 nm using a plate reader (Tecan Spark 10M; Tecan, Männedorf, Switzerland) at 25 °C under constant stirring as previously described [26]. Skeletal-muscle mitochondria (0.4–0.5 mg of mitochondrial protein/mL) were suspended in an incubation medium containing 210 mM mannitol, 70 mM sucrose, 1 mM KH_2_PO_4_, 50 μM arsenazo III, 10 μM EGTA, and 10 mM HEPES-KOH (pH 7.4.) and energized with 2.5 mM glutamate + 2.5 mM malate. After the addition of 50 μM CaCl_2_, the rate of Ca^2+^ uptake by mitochondria (nmol Ca^2+^ × min^−1^ × mg^−1^ of mitochondrial protein) was determined in the presence of 1 μM CsA. To determine the ability of mitochondria to retain Ca^2+^, 10 μM CaCl_2_ was added into the reaction medium (without CsA) successively, with an interval of 90 s. After several additions, external [Ca^2+^] increased, indicating a massive release of the ion from the organelles due to the opening of the MPT pore. The amount of Ca^2+^ released upon permeability transition (defined as Ca^2+^ capacity) was used as a measure of the MPT pore opening probability.

### 4.8. Electrophoresis and Immunoblotting of Mitochondrial Proteins

To prepare samples for the determination of the level of mitochondrial proteins, samples of isolated mitochondria (2 mg/mL) were placed in Eppendorf tubes, solubilized in Laemmli buffer, and heated at 95 °C for 3 min. Sample aliquots normalized by the protein concentration (10 µg of mitochondrial protein) were applied to the lanes of a 12.5% polyacrylamide gel and subjected to SDS-PAGE. After electrophoresis, the samples were transferred to a 0.45-µm nitrocellulose membrane (Amersham, Munich, Germany) for Western blot analysis. A PageRuler Prestained Protein Ladder from Thermo Scientific (Thermo Scientific, Waltham, MA USA) was used as a marker. After overnight blocking, the membrane was incubated with the appropriate primary antibody. Monoclonal rabbit anti-MCU (#14997), anti-CBARA/MICU1 antibody (#12524), and anti-ANT2/SLC25A5 (#14671) antibodies were obtained from Cell Signalling Technology Inc. (Danvers, MA, USA). The total OXPHOS Rodent WB Antibody Cocktail (ab110413), anti-VDAC1 (ab154856) and the polyclonal rabbit anti-CCDC109B (ab170715), anti-cyclophilin F (CypD) (ab64935), and anti-ANT1 (ab102032) antibodies were obtained from Abcam (Cambridge, UK). The immunoreactivity was detected using the appropriate secondary antibody conjugated to horseradish peroxidase (#7074, Cell Signaling technology Inc., Danvers, MA, USA). Peroxidase activity was detected with ECL chemiluminescence reagents (Pierce, Rockford, IL, USA). The relative levels of the detected proteins were visualized using a LI-COR system (LI-COR, Lincoln, NE, USA) and were normalized by the total protein concentration. Optical density measurements were performed by LI-COR Image Studio software.

### 4.9. Statistical Analysis

The data are expressed as the mean ± standard error of the mean (m ± SEM). Statistical analysis of the data was carried out using the GraphPad Prism version 8.0 software for Windows (GraphPad Software, San Diego, CA, USA). The statistical significance of differences between the experimental groups was evaluated using one-way repeated analysis of variance (ANOVA) followed by Turkey’s *post hoc* test. The differences were considered statistically significant at *p* < 0.05.

## 5. Conclusions

The results obtained in this work indicate that the effects of the glucocorticoid deflazacort used in DMD therapy may be associated with mitochondria. Deflazacort-induced molecular rearrangements in mitochondrial machinery, on the one hand, improve the functioning of organelles, and on the other hand, can cause the development of numerous side effects characteristic of this drug. Further study of the molecular mechanisms of action of this glucocorticoid is necessary to determine the optimal dose and dosage regimen for DMD management.

## Abbreviations

DMD	Duchenne muscular dystrophy
MPT	Mitochondrial permeability transition
Dfz	Deflazacort

## References

[1] Emery A.E. (1991). Population frequencies of inherited neuromuscular diseases-a world survey. Neuromuscul. Disord..

[2] Romitti P.A., Zhu Y., Puzhankara S., James K.A., Nabukera S.K., Zamba G.K., Ciafaloni E., Cunniff C., Druschel C.M., Mathews K.D. (2015). Prevalence of Duchenne and Beckermuscular dystrophies in the United States. Pediatrics.

[3] Ohlendieck K., Campbell K.P. (1991). Dystrophin-associated proteins are greatly reduced in skeletal muscle from mdx mice. J. Cell Biol..

[4] Turner P.R., Westwood T., Regen C.M., Steinhardt R.A. (1988). Increased protein degradation results from elevated free calcium levels found in muscle from mdx mice. Nature.

[5] Spencer M.J., Tidball J.G. (1992). Calpain concentration is elevated although net calcium-dependent proteolysis is suppressed in dystrophin-deficient muscle. Exp. Cell Res..

[6] Lindahl M., Backman E., Henriksson K.G., Gorospe J.R., Hoffman E.P. (1995). Phospholipase A2 activity in dystrophinopathies. Neuromuscul. Disord..

[7] Allen D.G., Whitehead N.P., Froehner S.C. (2016). Absence of dystrophin disrupts skeletal muscle signaling: Rles of Ca^2+^, reactive oxygen species, and nitric oxide in the development of muscular dystrophy. Physiol. Rev..

[8] Hess J. (1965). Phosphorylase activity and glycogen, glucose-6-phosphate, and lactic acid content of human skeletal muscle in various myopathies. J. Lab. Clin. Med..

[9] Chi M.M.Y., Hintz C.S., McKee D., Felder S., Grant N., Kaiser K.K., Lowry O.H. (1987). Effect of Duchenne muscular dystrophy on enzymes of energy metabolism in individual muscle fibers. Metabolism.

[10] Chinet A., Even P., Decrouy A. (1994). Dystrophin-dependent efficiency of metabolic pathways in mouse skeletal muscles. Experientia.

[11] Van Bennekom C., Oerlemans F.T., Kulakowski S., De Bruyn C.H. (1984). Enzymes of purine metabolism in muscle specimens from patients with Duchenne-type muscular dystrophy. Adv. Exp. Med. Biol..

[12] Ge Y., Molloy M.P., Chamberlain J.S., Andrews P.C. (2003). Proteomic analysis of mdx skeletal muscle: Great reduction of adenylate kinase 1 expression and enzymatic activity. Proteomics.

[13] Bonsett C., Rudman A. (1984). Duchenne’s muscular dystrophy: A tissue culture perspective. Indiana Med..

[14] Kuznetsov A.V., Winkler K., Wiedemann F., Von Bossanyi P., Dietzmann K., Kunz W.S. (1998). Impaired mitochondrial oxidative phosphorylation in skeletal muscle of the dystrophin-deficient mdx mouse. Mol. Cell Biochem..

[15] Rybalka E., Timpani C.A., Cooke M.B., Williams A.D., Hayes A. (2014). Defects in mitochondrial ATP synthesis in dystrophindeficient Mdx skeletal muscles may be caused by complex I insufficiency. PLoS ONE.

[16] Hughes M.C., Ramos S.V., Turnbull P.C., Rebalka I.A., Cao A., Monaco C.M.F., Varah N.E., Edgett B.A., Huber J.S., Tadi P. (2019). Early myopathy in Duchenne muscular dystrophy is associated with elevated mitochondrial H2O2 emission during impaired oxidative phosphorylation. J. Cachexia Sarcopenia Muscle.

[17] Ronzoni E., Wald S., Berg L., Ramsey R. (1958). Distribution of high energy phosphate in normal and dystrophic muscle. Neurology.

[18] Vignos P., Warner J. (1963). Glycogen, creatine and high energy phosphate in human muscle disease. J. Lab. Clin. Med..

[19] Cole M., Rafael J., Taylor D., Lodi R., Davies K., Styles P. (2002). A quantitative study of bioenergetics in skeletal muscle lacking utrophin and dystrophin. Neuromuscul. Disord..

[20] Shuttlewood R., Griffiths J. (1982). The purine nucleotide profile in mouse, chicken and human dystrophic muscle: An abnormal ratio of inosine plus adenine nucleotides to guanine nucleotides. Clin. Sci..

[21] Godin R., Daussin F., Matecki S., Li T., Petrof B.J., Burelle Y. (2012). Peroxisome proliferator activated receptor γ coactivator 1-α gene transfer restores mitochondrial biomass and improves mitochondrial calcium handling in post-necrotic mdx mouse skeletal muscle. J. Physiol..

[22] Kennedy T.L., Moir L., Hemming S., Edwards B., Squire S., Davies K., Guiraud S. (2017). Utrophin influences mitochondrial pathology and oxidative stress in dystrophic muscle. Skelet. Muscle.

[23] Vila M.C., Rayavarapu S., Hogarth M.W., Van der Meulen J.H., Horn A., Defour A., Takeda S., Brown K.J., Hathout Y., Nagaraju K. (2017). Mitochondria mediate cell membrane repair and contribute to Duchenne muscular dystrophy. Cell Death Differ..

[24] Jung C., Martins A.S., Niggli E., Shirokova N. (2008). Dystrophic cardiomyopathy: Amplification of cellular damage by Ca^2+^ signalling and reactive oxygen species-generating pathways. Cardiovasc. Res..

[25] Ascah A., Khairallah M., Daussin F., Bourcier-Lucas C., Godin R., Allen B.G., Petrof B.J., Rosiers C.D., Burelle Y. (2011). Stress-induced opening of the permeability transition pore in the dystrophin-deficient heart is attenuated by acute treatment with sildenafil. Am. J. Physiol. Heart Circ. Physiol..

[26] Dubinin M.V., Talanov E.Y., Tenkov K.S., Starinets V.S., Mikheeva I.B., Sharapov M.G., Belosludtsev K.N. (2020). Duchenne muscular dystrophy is associated with the inhibition of calcium uniport in mitochondria and an increased sensitivity of the organelles to the calcium-induced permeability transition. Biochim. Biophys. Acta Mol. Basis Dis..

[27] Morine K.J., Bish L.T., Pendrak K., Sleeper M.M., Barton E.R., Sweeney H.L. (2010). Systemic myostatin inhibition via liver-targeted gene transfer in normal and dystrophic mice. PLoS ONE.

[28] Mendell J.R., Sahenk Z., Lehman K., Nease C., Lowes L.P., Miller N.F., Iammarino M.A., Alfano L.N., Nicholl A., Al-Zaidy S. (2020). Assessment of systemic delivery of rAAVrh74.MHCK7.micro-dystrophin in children with Duchenne muscular dystrophy: A nonrandomized controlled trial. JAMA Neurol..

[29] Markham A., Bryson H.M. (1995). Deflazacort. A review of its pharmacological properties and therapeutic efficacy. Drugs.

[30] Bylo M., Farewell R., Coppenrath V.A., Yogaratnam D. (2020). A review of deflazacort for patients with Duchenne muscular dystrophy. Ann. Pharmacother..

[31] Bello L., Gordish-Dressman H., Morgenroth L.P., Henricson E.K., Duong T., Hoffman E.P., Cnaan A., McDonald C.M. (2015). Prednisone/prednisolone and deflazacort regimens in the CINRG Duchenne natural history study. Neurology.

[32] Griggs R.C., Miller J.P., Greenberg C.R., Fehlings D.L., Pestronk A., Mendell J.R., Moxley R.T., King W., Kissel J.T., Cwik V. (2016). Efficacy and safety of deflazacort vs prednisone and placebo for Duchenne muscular dystrophy. Neurology.

[33] McDonald C.M., Henricson E.K., Abresch R.T., Duong T., Joyce N.C., Hu F., Clemens P.R., Hoffman E.P., Cnaan A., Gordish-Dressman H. (2018). Long-term effects of glucocorticoids on function, quality of life, and survival in patients with Duchenne muscular dystrophy: A prospective cohort study. Lancet.

[34] Hanaoka B.Y., Peterson C.A., Horbinski C., Crofford L.J. (2012). Implications of glucocorticoid therapy in idiopathic inflammatory myopathies. Nat. Rev. Rheumatol..

[35] Schakman O., Gilson H., Kalista S., Thissen J. (2009). Mechanisms of muscle atrophy induced by glucocorticoids. Hormone Res..

[36] Quattrocelli M., Barefield D.Y., Warner J.L., Vo A.H., Hadhazy M., Earley J.U., Demonbreun A.R., McNally E.M. (2017). Intermittent glucocorticoid steroid dosing enhances muscle repair without eliciting muscle atrophy. J. Clin. Investig..

[37] Hammers D.W., Hart C.C., Patsalos A., Matheny M.K., Wright L.A., Nagy L., Sweeney H.L. (2020). Glucocorticoids counteract hypertrophic effects of myostatin inhibition in dystrophic muscle. JCI Insight.

[38] Raffaello A., De Stefani D., Sabbadin D., Teardo E., Merli G., Picard A., Checchetto V., Moro S., Szabo I., Rizzuto R. (2013). The mitochondrial calcium uniporter is a multimer that can include a dominant-negative pore-forming subunit. EMBO J..

[39] Sancak Y., Markhard A.L., Kitami T., Kovacs-Bogdan E., Kame K.J., Udeshi N.D., Carr S.A., Chaudhuri D., Clapham D.E., Li A.A. (2013). EMRE is an essential component of the mitochondrial calcium uniporter complex. Science.

[40] Mallilankaraman K., Cardenas C., Doonan P.J., Chandramoorthy H.C., Irrinki K.M., Golenar T., Csordas G., Madireddi P., Yang J., Muller M. (2012). MCUR1 is an essential component of mitochondrial Ca^2+^ uptake that regulates cellular etabolism. Nat. Cell Biol..

[41] Paillard M., Csordás G., Szanda G., Golenár T., Debattisti V., Bartok A., Wang N., Moffat C., Seifert E.L., Spät A. (2017). Tissue-specific mitochondrial decoding of cytoplasmic Ca^2+^ signals is controlled by the stoichiometry of MICU1/2 and MCU. Cell Rep..

[42] Giorgi C., Marchi S., Pinton P. (2018). The machineries, regulation and cellular functions of mitochondrial calcium. Nat. Rev. Mol. Cell Biol..

[43] Belosludtsev K.N., Dubinin M.V., Belosludtseva N.V., Mironova G.D. (2019). Mitochondrial Ca^2+^ transport: Mechanisms, molecular structures, and role in cells. Biochemistry.

[44] Belosludtsev K.N., Talanov E.Y., Starinets V.S., Agafonov A.V., Dubinin M.V., Belosludtseva N.V. (2019). Transport of Ca^2+^ and Ca^2+^-dependent permeability transition in rat liver mitochondria under the streptozotocin-induced type I diabetes. Cells.

[45] Kaludercic N., Scorrano L. (2019). MCUB hearts mitochondria in sickness, less in health. Circulation.

[46] Lambert J.P., Luongo T.S., Tomar D., Jadiya P., Gao E., Zhang X., Lucchese A.M., Kolmetzky D.W., Shah N.S., Elrod J.W. (2019). MCUB regulates the molecular composition of the mitochondrial calcium uniporter channel to limit mitochondrial calcium overload during stress. Circulation.

[47] Bhosale G., Sharpe J.A., Koh A., Kouli A., Szabadkai G., Duchen M.R. (2017). Pathological consequences of MICU1 mutations on mitochondrial calcium signalling and bioenergetics. Biochim. Biophys. Acta Mol. Cell Res..

[48] Dubinin M.V., Talanov E.Y., Tenkov K.S., Starinets V.S., Mikheeva I.B., Belosludtsev K.N. (2020). Transport of Ca^2+^ and Ca^2+^-dependent permeability transition in heart mitochondria in the early stages of Duchenne muscular dystrophy. Biochim. Biophys. Acta Bioenerg..

[49] Schiavone M., Zulian A., Menazza S., Petronilli V., Argenton F., Merlini L., Sabatelli P., Bernardi P. (2017). Alisporivir rescues defective mitochondrial respiration in Duchenne muscular dystrophy. Pharmacol. Res..

[50] Rasola A., Bernardi P. (2011). Mitochondrial permeability transition in Ca(2+)-dependent apoptosis and necrosis. Cell Calcium..

[51] Jonas E.A., Porter G.A., Beutner G., Mnatsakanyan N., Alavian K.N. (2015). Cell death disguised: The mitochondrial permeability transition pore as the c-subunit of the F(1)F(O) ATP synthase. Pharmacol. Res..

[52] Briston T., Selwood D.L., Szabadkai G., Duchen M.R. (2019). Mitochondrial permeability transition: A molecular lesion with multiple drug targets. Trends Pharmacol. Sci..

[53] Neginskaya M.A., Solesio M.E., Berezhnaya E.V., Amodeo G.F., Mnatsakanyan N., Jonas E.A., Pavlov E.V. (2019). ATP synthase c-subunit-deficient mitochondria have a small cyclosporine A-sensitive channel, but lack the permeability transition pore. Cell Rep..

[54] Bonora M., Pinton P. (2019). A New current for the mitochondrial permeability transition. Trends Biochem. Sci..

[55] Archer J.D., Vargas C.C., Anderson J.E. (2006). Persistent and improved functional gain in mdx dystrophic mice after treatment with L-arginine and deflazacort. FASEB J..

[56] Symons A.M., Lewis D.A., Ancill R.J. (1974). The actions of anti-inflammatory steroids on isolated rat liver mitochondrial function. J. Steroid Biochem..

[57] Brouilly N., Lecroisey C., Martin E., Pierson L., Mariol M.C., Qadota H., Labouesse M., Streichenberger N., Mounier N., Gieseler K. (2015). Ultra-structural time-course study in the C. elegans model for Duchenne muscular dystrophy highlights a crucial role for sarcomere-anchoring structures and sarcolemma integrity in the earliest steps of the muscle degeneration process. Hum. Mol. Genet..

[58] Hewitt J.E., Pollard A.K., Lesanpezeshki L., Deane C.S., Gaffney C.J., Etheridge T., Szewczyk N.J., Vanapalli S.A. (2018). Muscle strength deficiency and mitochondrial dysfunction in a muscular dystrophy model of Caenorhabditis elegans and its functional response to drugs. Dis. Model Mech..

[59] Passaquin A.C., Lhote P., Rüegg U.T. (1998). Calcium influx inhibition by steroids and analogs in C2C12 skeletal muscle cells. Br. J. Pharmacol..

[60] Karch J., Bround M.J., Khalil H., Sargent M.A., Latchman N., Terada N., Peixoto P.M., Molkentin J.D. (2019). Inhibition of mitochondrial permeability transition by deletion of the ANT family and CypD. Sci. Adv..

[61] Koshkin V., Bikopoulos G., Chan C.B., Wheeler M.B. (2004). The characterization of mitochondrial permeability transition in clonal pancreatic beta-cells. Multiple modes and regulation. J. Biol. Chem..

[62] Panov A., Dikalov S., Shalbuyeva N., Hemendinger R., Greenamyre J.T., Rosenfeld J. (2007). Species- and tissue-specific relationships between mitochondrial permeability transition and generation of ROS in brain and liver mitochondria of rats and mice. Am. J. Phys. Cell Physiol..

[63] Endlicher R., Kriváková P., Lotkova H., Milerová M., Drahota Z., Cervinková Z. (2009). Tissue specific sensitivity of mitochondrial permeability transition pore to Ca^2+^ ions. Acta Med. (Hradec Kralove).

[64] Laker R.C., Taddeo E.P., Akhtar Y.N., Zhang M., Hoehn K.L., Yan Z. (2016). The mitochondrial permeability transition pore regulator cyclophilin D exhibits tissuespecific control of metabolic homeostasis. PLoS ONE.

[65] Graham B.H., Waymire K.G., Cottrell B., Trounce I.A., MacGregor G.R., Wallace D.C. (1997). A mouse model for mitochondrial myopathy and cardiomyopathy resulting from a deficiency in the heart/muscle isoform of the adenine nucleotide translocator. Nat. Genet..

[66] Wu S., Sampson M.J., Decker W.K., Craigen W.J. (1999). Each mammalian mitochondrial outer membrane porin protein is dispensable: Effects on cellular respiration. Biochim. Biophys. Acta.

[67] Hyzewicz J., Tanihata J., Kuraoka M., Ito N., Miyagoe-Suzuki Y., Takeda S. (2015). Low intensity training of *mdx* mice reduces carbonylation and increases expression levels of proteins involved in energy metabolism and muscle contraction. Free Radic. Biol. Med..

[68] Neginskaya M.A., Strubbe J.O., Amodeo G.F., West B.A., Yakar S., Bazil J.N., Pavlov E.V. (2020). The very low number of calcium-induced permeability transition pores in the single mitochondrion. J. Gen. Physiol..

[69] Colell A., Garcia-Ruiz C., Lluis M., Coll O., Mari M., Fernandez-Chaca J.C. (2003). Cholesterol impairs the adenine nucleotide translocator-mediated mitochondrial permeability transition through altered membrane fluidity. J. Biol. Chem..

[70] Guiraud S., Davies K.E. (2017). Pharmacological advances for treatment in Duchenne muscular dystrophy. Curr. Opin. Pharmacol..

[71] Peterson J.M., Bakkar N., Guttridge D.C. (2011). NF-kappaB signaling in skeletal muscle health and disease. Curr. Top. Dev. Biol..

[72] Rosenberg A.S., Puig M., Nagaraju K., Hoffman E.P., Vil-lalta S.A., Rao V.A., Wakefield L.M., Woodcock J. (2015). Immune-mediated pathology in Duchenne muscular dystrophy. Sci. Transl. Med..

[73] Peterson J.M., Wang D.J., Shettigar V., Roof S.R., Canan B.D., Bakkar N., Shintaku J., Gu J.M., Little S.C., Ratnam N.M. (2018). NF-κB inhibition rescues cardiac function by remodeling calcium genes in a Duchenne muscular dystrophy model. Nat. Commun..

[74] Guseva N.V., Taghiyev A.F., Sturm M.T., Rokhlin O.W., Cohen M.B. (2004). Tumor necrosis factor-related apoptosis-inducing ligand-mediated activation of mitochondria-associated nuclear factor-kappaB in prostatic carcinoma cell lines. Mol. Cancer Res..

[75] Cogswell P.C., Kashatus D.F., Keifer J.A., Guttridge D.C., Reuther J.Y., Bristow C., Roy S., Nicholson D.W., Baldwin A.S. (2003). NF-kappa B and I kappa B alpha are found in the mitochondria. Evidence for regulation of mitochondrial gene expression by NF-kappa B. J. Biol. Chem..

[76] Nan J., Hu H., Sun Y., Zhu L., Wang Y., Zhong Z., Zhao J., Zhang N., Wang Y., Wang Y. (2017). TNFR2 stimulation promotes mitochondrial fusion via Stat3- and NF-kB dependent activation of OPA1 expression. Circ. Res..

[77] Liu H., Ma Y., Pagliari L.J., Perlman H., Yu C., Lin A., Pope R.M. (2004). TNF-alpha-induced apoptosis of macrophages following inhibition of NF-kappa B: A central role for disruption of mitochondria. J. Immunol..

[78] Psarra A.M., Sekeris C.E. (2008). Nuclear receptors and other nuclear transcription factors in mitochondria: Regulatory molecules in a new environment. Biochim. Biophys. Acta.

[79] Calvo S.E., Clauser K.R., Mootha V.K. (2016). MitoCarta2.0: An updated inventory of mammalian mitochondrial proteins. Nucleic Acids Res..

[80] Deacon R.M. (2013). Measuring motor coordination in mice. J. Vis. Exp..

[81] Frezza C., Cipolat S., Scorrano L. (2007). Organelle isolation: Functional mitochondria from mouse liver, muscle and cultured fibroblasts. Nat. Protoc..

[82] Chance B., Williams G.R. (1955). Respiratory enzymes in oxidative phosphorylation. I. Kinetics of oxygen utilization. J. Biol. Chem..

